# A novel multi-scale network intrusion detection model with transformer

**DOI:** 10.1038/s41598-024-74214-w

**Published:** 2024-10-05

**Authors:** Chiming Xi, Hui Wang, Xubin Wang

**Affiliations:** School of Computer Science and Information Engineering, Shanghai Institute of Technology, Shanghai, 201418 China

**Keywords:** Intrusion detection system, Transformer, Multi-scale data, Deep learning, Computer science, Information technology, Software

## Abstract

Network is an essential tool today, and the Intrusion Detection System (IDS) can ensure the safe operation. However, with the explosive growth of data, current methods are increasingly struggling as they often detect based on a single scale, leading to the oversight of potential features in the extensive traffic data, which may result in degraded performance. In this work, we propose a novel detection model utilizing multi-scale transformer namely IDS-MTran. In essence, the collaboration of multi-scale traffic features broads the pattern coverage of intrusion detection. Firstly, we employ convolution operators with various kernels to generate multi-scale features. Secondly, to enhance the representation of features and the interaction between branches, we propose Patching with Pooling (PwP) to serve as a bridge. Next, we design multi-scale transformer-based backbone to model the features at diverse scales, extracting potential intrusion trails. Finally, to fully capitalize these multi-scale branches, we propose the Cross Feature Enrichment (CFE) to integrate and enrich features, and then output the results. Sufficient experiments show that compared with other models, the proposed method can distinguish different attack types more effectively. Specifically, the accuracy on three common datasets NSL-KDD, CIC-DDoS 2019 and UNSW-NB15 has all exceeded 99%, which is more accurate and stable.

## Introduction

The network is becoming indispensable in people’s life and work, gradually permeating every aspect. Consequently, concerns about security are increasingly being raised. Given the rapid growth of the internet and the explosion of usage, any malicious intrusion or attack on network vulnerability can lead to a serious disaster ^1^. Intrusion Detection System (IDS) is a security tool used to monitor computer networks for suspicious activity, which aims to identify, log and alert potential security threats. Nowadays, with the volume of data still surging, IDS that enables the network to avoid attacks and effectively reduce economic losses is taken ever more seriously ^2^.

Traditionally, signature-based approaches have been important for a long time. However, with the explosion of data, signature database must be updated frequently to keep up with evolving intrusion tactics. Competent in pattern recognition, deep learning-based IDS is increasingly favored and gradually supplanting signature-based approaches^3^. For instance, Convolutional Neural Networks (CNN)^4,5^, Recurrent Neural Networks (RNN)^6^, and Long Short-Term Memory Neural Networks (LSTM)^7^ are widely used for IDS. However, such data-driven models also have limitations, they often struggle with specific types of attacks as the variations in traffic features are sometimes subtle and are often overlooked^8^.

How to extract key attack features is the most important issue in anomaly-based IDS^9^. In recent years, Transformer^10^ that continues to show State-Of-The-Art (SOTA) performance in many fields has also been gradually applied to IDS with favorable performance^11,12^. Benefiting from the powerful self-attention mechanism, such models can analyze complex network traffic in a more in-depth manner, thus effectively discern correlations in sequence data and modeling globally in traffic analysis. However, some problems related to noise components and minor features in traffic data still constrain the performance^13^, and need to be tackled critically.

Upon observation, prevalent methods often process with single-scale traffic data, which ignore the information richness of features at different scales. Typically, multi-scale data is considered to cover a more comprehensive range of features, and the utilization of multi-scale data has proven to be an effective performance improvement method in many fields^14^.However, it remains insufficiently explored in the context of IDS.

Based on the discussions above, this paper propose IDS-MTran, a novel multi-scale pipeline based on Transformer. It is designed to efficiently incorporate features at different scales to improve the detection, as well as utilizing the excellent global modeling capability of Transformer. In essence, the collaboration of multi-scale traffic features can broad the pattern coverage of intrusion detection, thus improve the performance. Initially, IDS-MTran produces features at different scales from existing data using different operators as the basis for detection. Subsequently, it enhances these representations and highlights the scale advantage through the newly proposed PwP (Patching with Pooling) module, which aims to interact features at different levels and weaken the noise to better recognize attack types. Afterwards, the three Transformer-based backbone networks output the feature representations corresponding to each branch. On the basis of current multi-scale architecture, especially those well-performed models, the effective handling of multi-scale features is a crucial issue. For IDS-Mtran, it incorporates different through the newly proposed CFE (Cross Feature Enrichment) module, which enriches the features received through interactions and combines them organically, as well as predicts the final results.

Finally, we conduct comprehensive experiments on the commonly used NSL-KDD,CIC-DDoS 2019 and UNSW-NB15 datasets, and the results show that the proposed IDS-MTran is an effective and advanced method, particularly showing the SOTA performance in the identification of specific attack categories. Furthermore, ablation experiments validate the effectiveness of the multi-scale design.

The structure of this paper is as follows. Section "Related work" presents the related work with IDS. Section "Methodology" presents our method in detail, including the optimization process. Section "Experiments" presents the experiment and results with detailed analysis. Finally, we conclude the paper in section "Conclusions".

## Related work

Typically, IDS can be divided into two categories: signature-based and anomaly-based^15,16^. The former relies on traffic signatures, necessitating continual updates to the latest signature database. It is effective for detecting known types of attacks, but incapable of identifying new and unknown types. The latter is assessed by evaluating the deviation between monitored and normal traffic, while it excels in detecting unknown attacks and is prevalent in contemporary IDS systems, it is prone to false alarms, and its accuracy requires enhancement^17^.

### Signature-based methods

Signature intrusion detection systems (SIDS) employ pattern matching methodologies to identify known attacks. These systems are alternatively referred to as Knowledge-based Detection Systems or Misuse Detection Systems^18^.Raiah et al.^19^ have developed a trust-aware signature-based IDS that utilizes trust tables to detect potential intrusions in the MANET nodes,which achieved a minimum latency of 0.00434 second, low energy consumption of 9.933 joules, high detection rate of 0.623, and throughput of 0.642 packets per second. Both He et al.^20^ and Sutskever et al.^21^ developed signature-based routing protocols to detect Sybil attacks in the Internet of Things. Despite they are effective at detecting known intrusions, they are increasingly inadequate for today’s complex and dynamic network environments.

### Anomaly-based methods

Among anomaly-based approaches, machine learning has gained widespread recognition for its adaptive and powerful data handling capabilities, addressing contemporary IDS requirements^22^. Some classical models are widely used in IDS. For instance, Hota et al.^23^ combined feature engineering and the C4.5 decision tree technique, taking accuracy to new heights, Kabir et al.^24^ proposed optimum allocation-based least square support vector machine (OA-LS-SVM) for IDS, achieving better results in terms of efficiency and accuracy. To date, these models still play an important role. For instance, Mahbooba et al.^25^ employed decision trees to address non-linear relationships in intrusion detection data, thereby obviating the need for excessive pre-processing of data and enhancing model detection efficiency. Zhang et al.^26^ employed weighted PCA to mitigate the impact of data contamination and enhance the accuracy of the assay. The conventional machine learning methods primarily focus on shallow learning, which emphasizes feature engineering and selection. Mohammad et al. ^27^ proposed an automatic clustering algorithm based on consistency and separability for optimizing attack clustering in intrusion detection systems. Combining Artificial Bee Colony Algorithm (ABC), Particle Swarm Optimization (PSO) and Differential Evolution (DE) methods, the algorithm performs well in terms of optimization of the number of clusters, the number of evaluation functions and accuracy. As the dataset size increases, shallow learning becomes inadequate for intelligent analysis due to its requirement for high-dimensional learning with substantial volumes of data.

Deep learning, an end-to-end approach, is increasingly favored among anomaly-based detection techniques^28,29^. Deep learning-based IDS offers considerable benefits, making IDS more robust and intelligent. For example, Li et al.^30^ converted feature data to a grayscale graph and proposed multi-CNN fusion model, outperforming traditional machine learning methods. Ding et al. ^31^ proposed a CNN-based IDS model for multi-category classification experiments using the NSL-KDD dataset. The study shows that deep learning has significant advantages in large-scale data feature extraction and provides a new research direction for intrusion detection.

In addition, Artificial Neural Networks (ANNs) have also achieved significant results in anomaly detection. Rahim et al. ^32^ screened features through the cuttlefish algorithm and evaluated the performance of different feature combinations using ANNs. The experimental results show that 13 feature combinations can efficiently detect almost all attacks, significantly improving the accuracy rate. Bhupendra et al. ^33^ evaluated the NSL-KDD dataset through ANNs in anomaly traffic detection, and the results show that the detection rates of intrusion detection and attack type classification are 81.2% and 79.9%, respectively, which further validates the effectiveness of ANN in improving the detection accuracy.

Notably, RNNs are often better suited than CNNs to detect intrusion as traffic data generally exhibits sequential nature. For instance, Kasongo^34^ incorporated different types of Recurrent Neural Networks (RNN), namely Long-Short-Term Memory (LSTM), Gated Recurrent Units (GRU) and Simple RNN, with an XGBoost-based feature selection algorithm. The XGBoost-LSTM model performs best on the NSL-KDD dataset, while the XGBoost-Simple-RNN model achieves the most efficient performance on the UNSW-NB15 dataset. Oliveira et al.^35^ proposed a LSTM-based method, the experimental results show that the LSTM network has excellent reliability in effectively capturing sequential patterns in network traffic data, with an accuracy of 99.94% and an F1 score of 91.66%. Silivery et al.^36^ combined RNN, LSTM, and DNN to propose a hybrid network model that achieved quite good performance.

In recent years, Transformer^10^ continues to show SOTA performance in many fields. Various studies show its efficacy in processing sequential data, where the multi-head self-attention mechanism enables the network to capture contextual information from the entire sequence. This advanced model is also applied in IDS, exhibiting superior performance^11,12^. For example, Nguyen et al.^37^ proposed a transformer-based attention network (TAN) for an in-vehicle CAN bus, which is more efficient and powerful. Zhang et al.^38^ proposed a novel intrusion detection model that integrates CNN and Transformer, enabling the capture of both global correlations between packets and identification of local correlations associated with intrusions. Yang et al.^12^ proposed an intrusion detection model based on an improved vision transformer. The experiments conducted on the NSL-KDD dataset demonstrate that the model achieves an accuracy of 99.68%, a false alarm rate as low as 0.22%, and an recall rate of 99.57%.

Furthermore, researchers often leverage threat models to help security teams identify the attacks and vulnerabilities they are most likely to face and, in turn, more effectively configure and tune signature-based or anomaly-based intrusion detection systems ^39,40^.

## Methodology

**Fig. 1:**
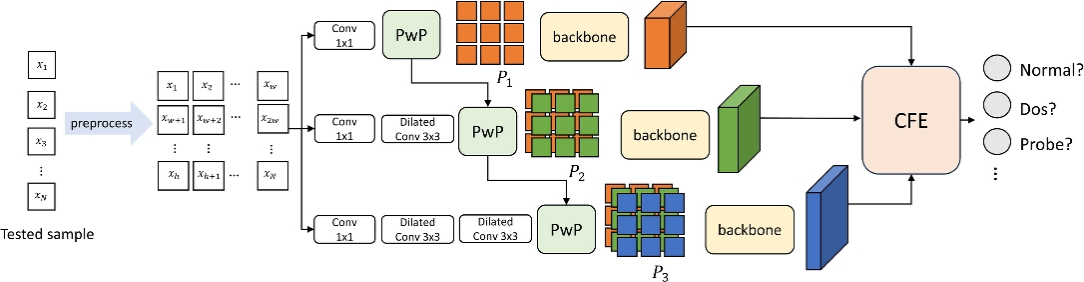
The overall structure of IDS-MTran.

Figure 1 shows the architecture of IDS-MTran, which extracts rich features from traffic data by creating multi-scale branches. It follows the end-to-end paradigm, where the inputs are pre-processed and then patched, and the patch groups are intersected to serve as inputs to the backbone. Features from different branches are organically integrated to obtain the result. The designed architecture is discussed in detail in this section.

### Preprocess

Given the traffic data to be tested $x={x_1,x_2,...,x_N}$, pre-processing is first performed, including digitization, addressing abnormal values, normalization, and matrixization:

1. Among the sample features, those containing character strings cannot be computed directly. Therefore, digitization is performed first, i.e., the strings are processed using one-hot coding. The specific encoding depends on the data.
2. Next, we need to find if there are outliers in the data. The handling relies on Gaussian distribution, determined by calculating the gap between the input samples and the mean of all data:
  1 $$\begin{aligned} f(x)=\frac{1}{\sqrt{2\pi }\sigma }\exp \left\{ -\frac{(x-\mu )^2}{2\sigma ^2}\right\} , \end{aligned}$$
  where $\sigma$ is the standard deviation, $\mu$ is the mean of the sample data, and *x* is the input data. Values with a gap of more than three times are determined to be an outlier.
3. To speed up optimization and training, the data needs to be normalized. The min–max method is leveraged to scale all features to the same range, as shown in Eq. 2:
  2 $$\begin{aligned} x^{\prime }=\frac{x-\min (x)}{\max (x)-\min (x)}. \end{aligned}$$
4. Matrixization aims to convert the input sequence into matrix for processing. For the flow sequence, it is converted into a two-dimensional matrix *X* of $h\times w$, as shown in Fig. 1. When *N* is not an integer multiple of *h*, the end of the data sequence is filled with 0.

### Multi-scale architecture

Confronted with extensive traffic data, the effective feature extraction is the key to detection. Existing methods tend to operate on a single data scale, ignoring the multi-scale information present in the data. In general, distinct data scales often encompass different information, e.g., lower-level features show basic structural details, while higher-level, more abstract features show overall trends. Upon the observation above, we construct a multi-scale architecture to improve the exploitation of traffic data.

As shown in Fig. 1, it contains three branches creating by different convolution kernels. For each, we first utilize $1\times 1$ to adjust the shape and channel. To exploit the potential feature, which is often deeper and more abstract, $3\times 3$ and $5\times 5$ kernel sizes are leveraged to the last two branches, respectively. Further, we use two parallel $3\times 3$ kernels instead of the $5\times 5$ one, since the parameter of the parallel is only 18 but not 25 and it brings a expanded receptive field. At the same time, all the larger convolutions are replaced with dilated convolution, which can increase the receptive field of the filter without increasing the parameters, thus making the feature extraction more comprehensive.

We postulate that higher-level features are effective at capturing macro patterns or trends in traffic data. Larger scales, on the other hand, are adept at discerning detailed features in traffic data, such as changes in the size of packets over a short period of time. With multi-scale network analysis, potential signs of intrusion can be identified from different perspectives and scales, providing a more comprehensive security analysis and enhances the detection sensitivity.

### Patching with Pooling

**Fig. 2:**
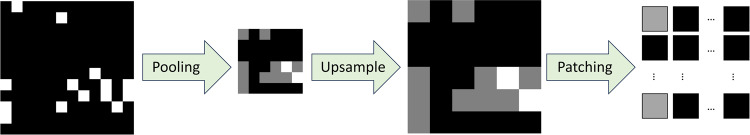
Illustration of PwP.

One of the reasons that traffic data is challenge to process is the low information density, where attack trails are often hidden in a large number of normal parameters to avoid detection systems. As shown in Fig. 1, we construct Patching with Pooling (PwP) for each branch, aiming at enhancing the key features from the background noise. Figure 2 shows its structure, which starts with the average pooling to reduce the data dimensions, helping to focus on a wider range of features and making the anomaly localization more easier. The up-sampling then re-introduces some of the detail that lost in the pooling, and simultaneously highlights interest features.

Consequently, we divide each feature map into $T=(h/s)\times (w/s)$ patches of size *s* to serve as the inputs. To preserve the organizational structure information during patch segmentation, we propose fusing groups of patches between different branches. As shown in Fig. 1, the low-level information is supplemented to the high-level features in a top-down manner. Where low-level features are considered as auxiliary and high-level features are considered as primary. The reason is that auxiliary features contain more detailed information, which helps to enrich the high-level information contained in the main features, thus obtaining richer and finer representation.

### Transformer-based backbone

Competent in sequential modeling, Transformer is widely used in intrusion detection. The pure attention mechanism allows it to focus on the most relevant parts of the data, and the parallel processing capability makes it more efficient when dealing with massive data.

**Fig. 3:**
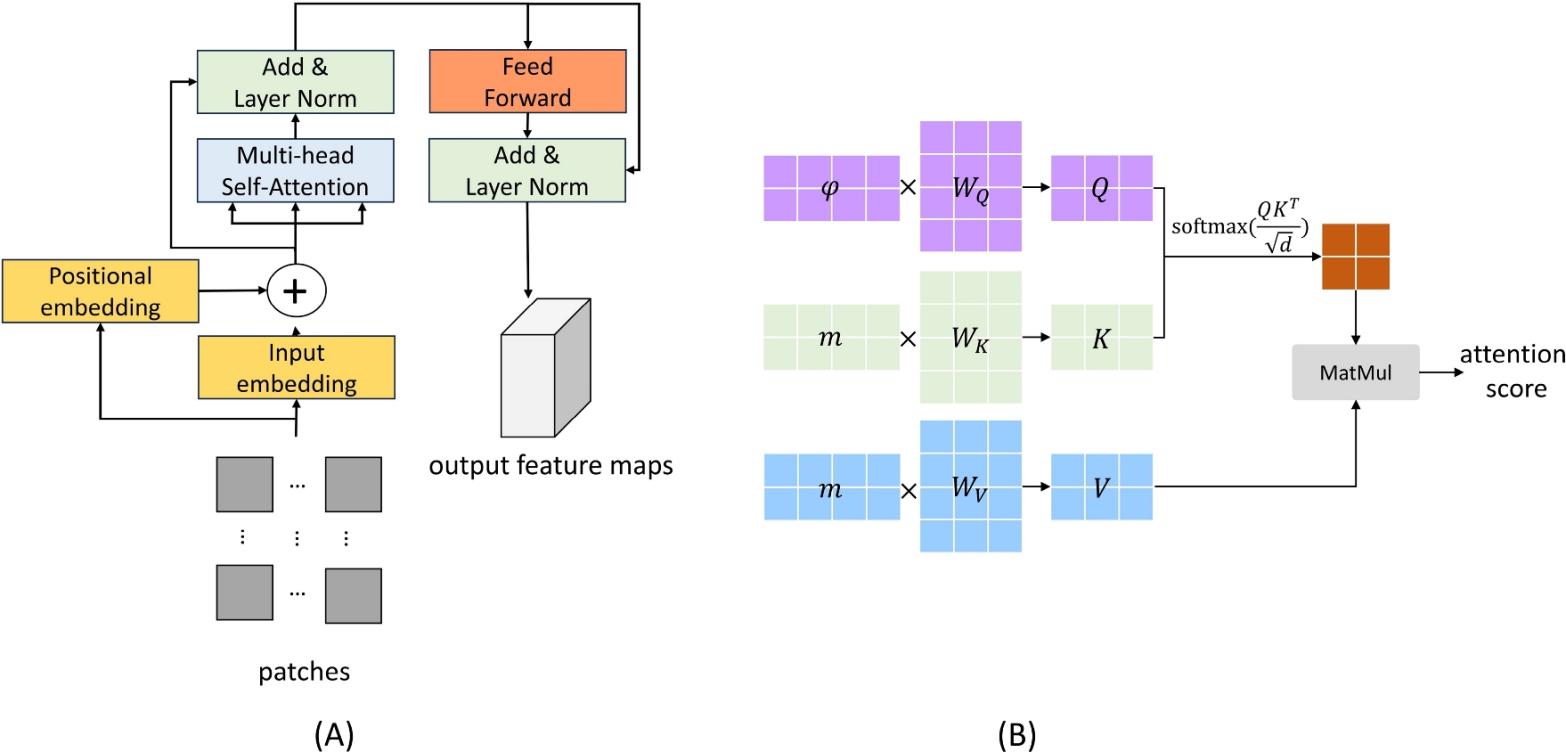
(**A**) The architecture of transformer-based backbone. (**B**) Illustration of the calculation of self-attention.

A Transformer model usually contains an encoder and a decoder to compress and recover the input sequence data, respectively. Considering that our framework requires only feature extraction and does not need to recover the dimension, we leverage the encoder as backbone to process multi-scale branches separately. Figure 3 shows the architecture.

Due to the lack of a looping structure for parallel computing, Transformers often do not naturally handle sequential information. Therefore, positional encoding is added to each patch as a supplement, which enables the model to be aware of the relative or absolute position in the original input sequence. We leverage the common cosine and sine functions to encode:

3 $$\begin{aligned} {\left\{ \begin{array}{ll} PE_{(pos,2i)}=\sin \left( \frac{pos}{10000}\right) \\ PE_{(pos,2i+1)}=\cos \left( \frac{pos}{10000}\right) & \end{array}\right. }, \end{aligned}$$

which are then summed with the embedding of the sequence to provide unique identifiers of different positions. It facilitates the model to learn the position information.

The self-attention mechanism, pivotal in the Transformer architecture, is designed to enhance sequence modeling by capturing dependencies regardless of their distance in the sequence. It operates using three matrices: $W_Q$ (Query), $W_K$ (Key), and $W_V$ (Value). Each element in the input sequence is transformed into these three representations. The query (*Q*) represents the part of sequence that is currently in focus, and the keys (*K*) act like tags to help identify the elements associated with the query. The value (*V*) represents the information that should be in focus when encoding a particular element. Self-attention calculates the attention score by comparing the similarity between *Q* and *K*, then weighted and summed with the *V* to form the final output for each element, as shown in Fig. 3.

Transformer uses the multi-head self-attention mechanism to perform multiple attention operators in parallel to help the model learn information from different representation sub-spaces. For each head, the attention is computed independently and the results are stitched together at the end:

4 $$\begin{aligned} MultiHead(Q,K,V)=Concat(head_1,...,head_h)W^O, \end{aligned}$$

where $head_i=\text {Attention}(QW_i^Q,KW_i^K,VW_i^V)$. By allowing the model to focus on multiple aspects of the sequence at the same time, this special mechanism significantly enhances the processing ability of Transformer, making it more efficient and accurate when dealing with complex sequential data.

Subsequently, the data stream is further processed through Layer Normalization and Feed-Forward Neural Network. Finally, by concatenating multiple such encoders, where the output of each becomes the input to the next layer, the backbone network of IDS-MTran is formed to encode the entire patch groups.

### Cross feature enrichment

The complexity and diversity of attacks make it difficult to accurately identify and defend against all types of attacks. Though the proposed method can extract traffic features at different scales, a comprehensive utilization strategy poses a significant consideration.

**Fig. 4:**
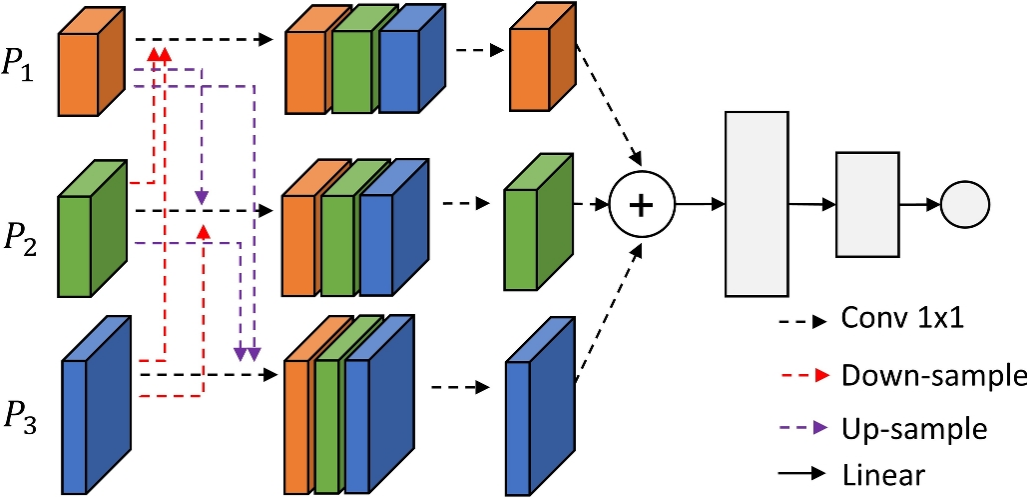
Architecture of the Cross Feature Enrichment.

To better leverage the features behind different scales, as shown in Fig. 4, we propose a novel Cross Feature Enrichment module to process. It is constructed to cross-enhance low-level and high-level information, which allows the model to learn richer features through cross-layer feature interactions. Specifically, features at three different scales are up-sampled and down-sampled into other branches, respectively, and then concated into new blended vectors. These composites simultaneously contain information at different perspectives, and we further down-sample them separately to distill the features. And this distillation integrates different perspectives, making branches more sensitive to attacks and improving the robustness.

Finally, we combine these enhanced features in the same dimension, and then output the final result using three linear layers. By adeptly combining information at different scales, CFE enables each branch to understand and respond to various attack types more thoroughly, thus making detection more comprehensive and accurate.

### Loss function

**Fig. 5:**
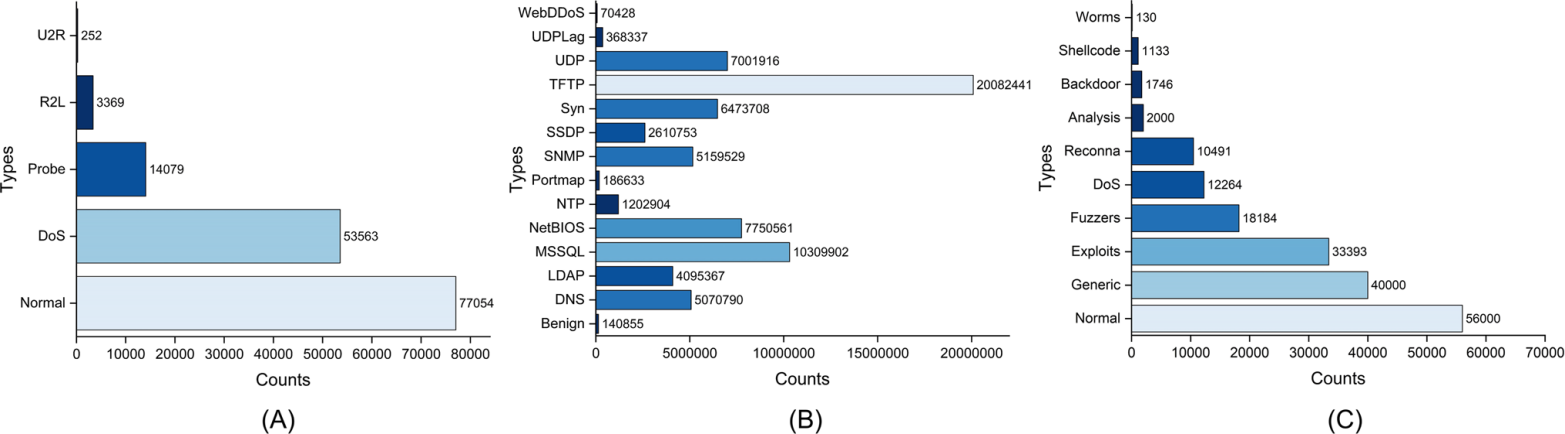
(**A**) NSL-KDD dataset sample distribution. (**B**) CIC-DDoS 2019 dataset sample distribution. (**C**) UNSW-NB15 dataset sample distribution.

Though IDS-MTran can effectively extract discriminative features from extensive traffic data, this presupposes an effective training process. Data imbalance is one of the most important considerations, as quantitatively dominant categories will guide the model to ignore those that are scarce. As shown in Fig. 5, the data used for training in intrusion detection tends to be extremely unbalanced, with the amount of normal traffic data being much higher than intrusion instances due to the fact that attack activity is harder to collect. Aiming at this, we adopt the focal loss^41^, which is widely used in computer vision to solve the data imbalance, to guide the training.

Focal Loss was originally designed to solve the problem of imbalance between foreground and background categories in target detection, and it is an improvement of the cross-entropy (CE). Given the predicted probability *p* and the ground truth label *y*, CE is defined as:

5 $$\begin{aligned} CE(p,y)={\left\{ \begin{array}{ll}-log(p),\mathrm {if~}y=1\\ -log(1-p),\textrm{otherwise}& \end{array}\right. }, \end{aligned}$$

which intuitively penalizes predictions that are inconsistent with true labels. By optimizing for overall loss using negative log-likelihood, the model is able to accurately predict the majority and easy-to-classify categories. However, anomalous traffic is often in the minority and hard to classify. Focal Loss relaxes this problem by focusing more on these samples located near the decision boundary in the feature space. Specifically, let $CE(p_t)=-\log (p_t)$, where

6 $$\begin{aligned} p_t={\left\{ \begin{array}{ll}p,\mathrm {if~}y=1\\ 1-p,\textrm{otherwise}& \end{array}\right. }, \end{aligned}$$

then focal loss can be written as:

7 $$\begin{aligned} FL(p_t)=-(1-p_t)^\gamma \log (p_t), \end{aligned}$$

where the $(1-p_t)^\gamma$ can be viewed as a modulating factor that reduces the weight of easy-to-classify samples and makes the model focus more on hard-to-classify ones. Specifically, $p_t$ will decrease if the sample belongs to latter, the loss will increase with $(1-p_t)^\gamma$, and the model will focus more on it. Additionally, a balancing factor $\alpha$ is introduced to further solve the imbalance:

8 $$\begin{aligned} FL(p_t)=-\alpha _t(1-p_t)^\gamma \log (p_t). \end{aligned}$$

By providing different weights for different categories, it helps to prevent the model from being overly biased in favor of the majority category in the case of extreme imbalance.

## Experiments

Beginning with a description of the data, environment and metrics used, this section presents the experiment results, including the comparative experiments and ablation studies.

### Datasets description

The NSL-KDD dataset^42^ is an improved version of the KDDCup99 dataset, developed by the National Institute of Standards and Technology (NIST) to facilitate research and evaluation of network intrusion detection. The dataset covers five network traffic types, including normal, DoS, Probe, U2R and R2L attacks, and contains a total of 148,517 data samples after processing the outliers. Figure 5 describes the distribution of each sample in the NSL-KDD dataset in detail. In the sample, the values corresponding to the three feature keys “Protocol type”, “Flag”, and “Service” are strings and need to be encoded.

The CIC-DDoS2019 dataset^43^ was developed by the Canadian Institute for Cybersecurity at the University of New Brunswick to investigate and evaluate the performance of distributed denial of service (DDoS) attack detection systems. It offers more comprehensive traffic features and exhibits a significantly high proportion of malicious traffic, comprising 7,040,987,392 instances, while only 140,855 records correspond to benign. The distribution of CIC-DDoS2019 is illustrated in Fig. 5.

The UNSW-NB15 dataset ^44^ was created by researchers at the Australian Centre for Cyber Security (ACCS) lab at the University of New South Wales (UNSW). This dataset contains raw network traffic data of monitored by TCP-Dump tool containing 2,540,044 realistic records. The dataset includes a wide variety of different types of network traffic, such as TCP, UDP, ICMP, and HTTP, the allocation of UNSW-NB15 is shown in Fig. 5, which also includes information on the source and destination of the traffic, as well as the time and duration of each packet.

The experiments are categorized into binary- and multiple- classification tasks, with the former aiming to discern whether traffic is malicious, and the latter being specific to the type of attack.

### Experimental environment and parameter settings

The hardware environment for the experiment is a workstation equipped with 64GB of RAM, Intel Core i7 13700k central processor, and Nvidia RTX 4090 24GB GPU. The software environment is Windows 11 operating system, python 3.8, PyTorch 1.12.1, Numpy 1.20.3, scikit-learn 1.1.2, and matplotlib 3.7.1.

The focal loss in section 3.6 is selected to train IDS-MTran, Adam optimizer is used to assist in training where $\beta _1=0.99$ and $\beta _2=0.9999$. The initial learning rate is set to 0.001, the batch size is set to 512, and the target epoch for training is 100 and the early stop strategy is applied. Note that for the detailed architecture of Transformer-based backbone, please refer to ^45^.

### Predictive model evaluation metrics

**Fig. 6:**
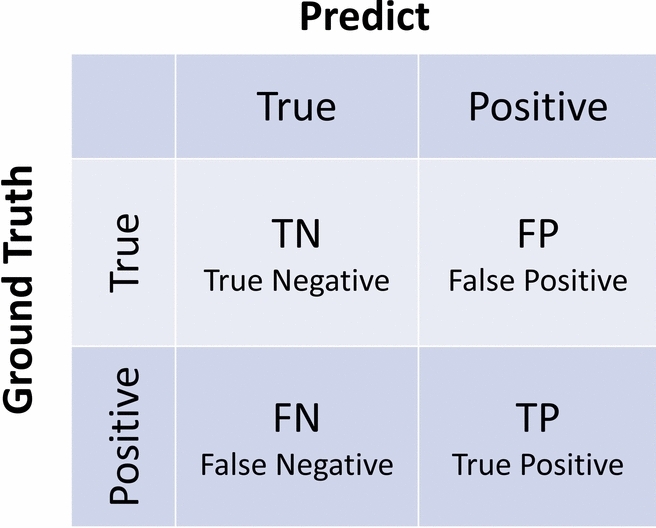
Illustration of the confusion matrix.

The predictive model is evaluated by a confusion matrix, which consists of four components as shown in Fig. 6 : TP: the instance is correctly identified as positive; FP: the instance is incorrectly identified as positive despite being negative; TN: the instance is correctly identified as negative; FN: the instance is incorrectly identified as negative despite being positive.

Consequently, four widely-used metrics-Accuracy, Precision, Recall, and F1 Score are selected. Accuracy is one of the most intuitive manifestations of the model’s performance:

9 $$\begin{aligned} Accuracy=\frac{TP+TN}{TP+FN+FP+TN}. \end{aligned}$$

Precision shows how accurately the model predicts positive samples:

10 $$\begin{aligned} Precision=\frac{TP}{TP+FP}. \end{aligned}$$

Recall represents the model’s proficiency in identifying intrusion traffic:

11 $$\begin{aligned} Recall=\frac{TP}{TP+FN}. \end{aligned}$$

F1-Score considers both recall and precision, and is a commonly used metric for evaluating multi-classifier models:

12 $$\begin{aligned} F1-Score=2\times \frac{Precision\times Recall}{Precision+Recall}. \end{aligned}$$

### Comparative experiments

We first conduct comparative experiments on the three datasets NSL-KDD, CIC-DDoS 2019 and UNSW-NB15 to validate the advancement of IDS-MTran. As mentioned above, these datasets possess different characteristics, thus the SOTA methods are not the same, and we introduce them in the corresponding subsections. Among the competitors, some classical IDS methods are selected, including CNN (ResNet34^20^), RNN^46^, LSTM^47^ and ViT^45,48^. Finally, we conduct the comparative analysis of the detection efficiency.

#### Comparison results on NSL-KDD

Performing detection on NSL-KDD is a relatively simple task in these three datasets, as NSL-KDD has been well-studied in recent years and has been used as the baseline data for many IDS models. Thus, we perform comparison on the classical methods, and some SOTA methods optimized specifically for IDS, including the method proposed by Liu et al. ^49^, the ANN method proposed by Zakariah et al. ^50^ and the AE method proposed by Xu et al. ^51^. Note that as a long-standing challenge, there are a number of excellent works on this dataset, such as the study of Meena et al. ^52^. Therefore, we also report the results of several machine learning methods for comparison.

Table 1 reports the results of binary-classification and multiple-classification results for each model. For the binary one, IDS-MTran outperforms others with 99.25% accuracy, 99.07% precision, 99.02% recall, and 99.05% F1-score, showing excellent overall performance. The traditional CNN model performs the weakest, with 91.86% accuracy and 89.21% F1 score, which reflects its limitations in handling sequential data. On the contrary, RNN and LSTM, which are adept at processing sequence data, perform extremely well, but still not as well as ours. The ViT model performs the best among these competitors, demonstrating the advantages that the global dependency brings to intrusion detection. But its performance is still lower than the proposed multi-scale model due to the under-utilization of the features with the single scale.

**Table 1:** Quantitative results on NSL-KDD.

Method	Binary-classification				Five-classification			
	Accuracy	Precision	Recall	F1-score	Accuracy	Precision	Recall	F1-score
CNN	91.86	90.93	87.82	89.21	85.12	86.62	85.13	85.40
RNN	97.64	97.45	96.39	96.91	93.16	94.02	91.40	92.56
LSTM	98.71	98.32	98.41	98.36	95.51	95.38	94.44	94.85
ViT	98.79	98.63	98.26	98.44	97.80	97.45	97.83	97.62
Liu et al. ^49^	92.90	89.92	98.57	94.05	85.24	–	–	–
ANN ^50^	97.50	99.00	96.70	95.70	–	–	–	–
AE ^51^	90.61	86.83	98.43	92.26	–	–	–	–
Ours	**99.25**	**99.07**	**99.02**	**99.05**	**99.16**	**99.01**	**99.17**	**99.09**

The values are expressed in %, and the best one is in bold.

For the five-classification task that is more complex compared to the binary one, where the model not only has to detect the presence of intrusion but also accurately predict the specific type. The transition from binary- to five- classification degrades the performance of all models, reflecting the challenging nature of the task. As reported in Table 1, the accuracy of the CNN decreased from 91.86 to 85.12%, indicating its diminished efficacy in dealing with more complex sequence problems. The performance of RNN also decreased, with accuracy dropping from 97.64 to 93.16%, indicating it is not as efficient as simple classification. LSTM and ViT show high stability, they perform well and their performance is similar to the binary task, implying their good adaptability to complex tasks. Notably, as the multi-scale’s all-around capability in macro and micro, the proposed method shows no almost degradation, with an accuracy of 99.16%. Its excellent performance on different attack categories exhibits its significant advantage in multi-category problems. Table 2 reports the quantitative results specific to attack types.

**Table 2:** Quantitative results of our method specific to attack types.

	Accuracy	Precision	Recall	F1-score
Normal	99.22	98.74	98.39	98.56
Dos	99.90	99.79	99.83	99.81
Probe	99.64	99.38	99.22	99.30
U2R	99.73	98.90	99.38	99.14
R2L	99.82	98.24	99.03	98.63

The values are expressed in %.

**Fig. 7:**
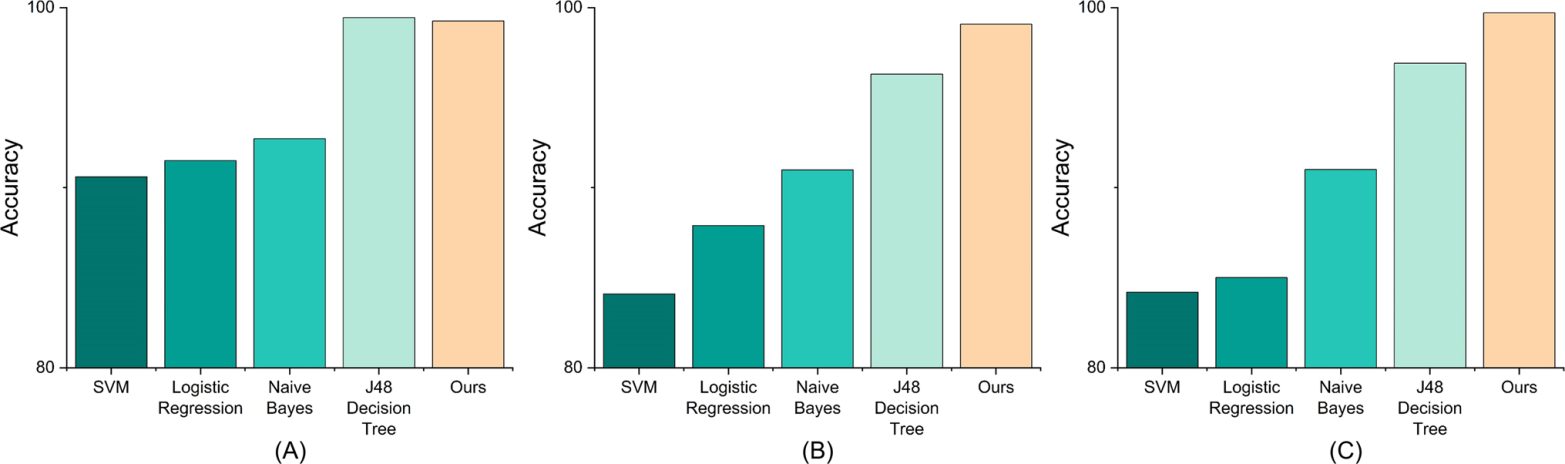
Comparison between IDS-MTran and several machine learning methods on (**A**) NSL-KDD, (**B**) CIC-DDoS 2019, and (**C**) UNSW-NB15.

**Fig. 8:**
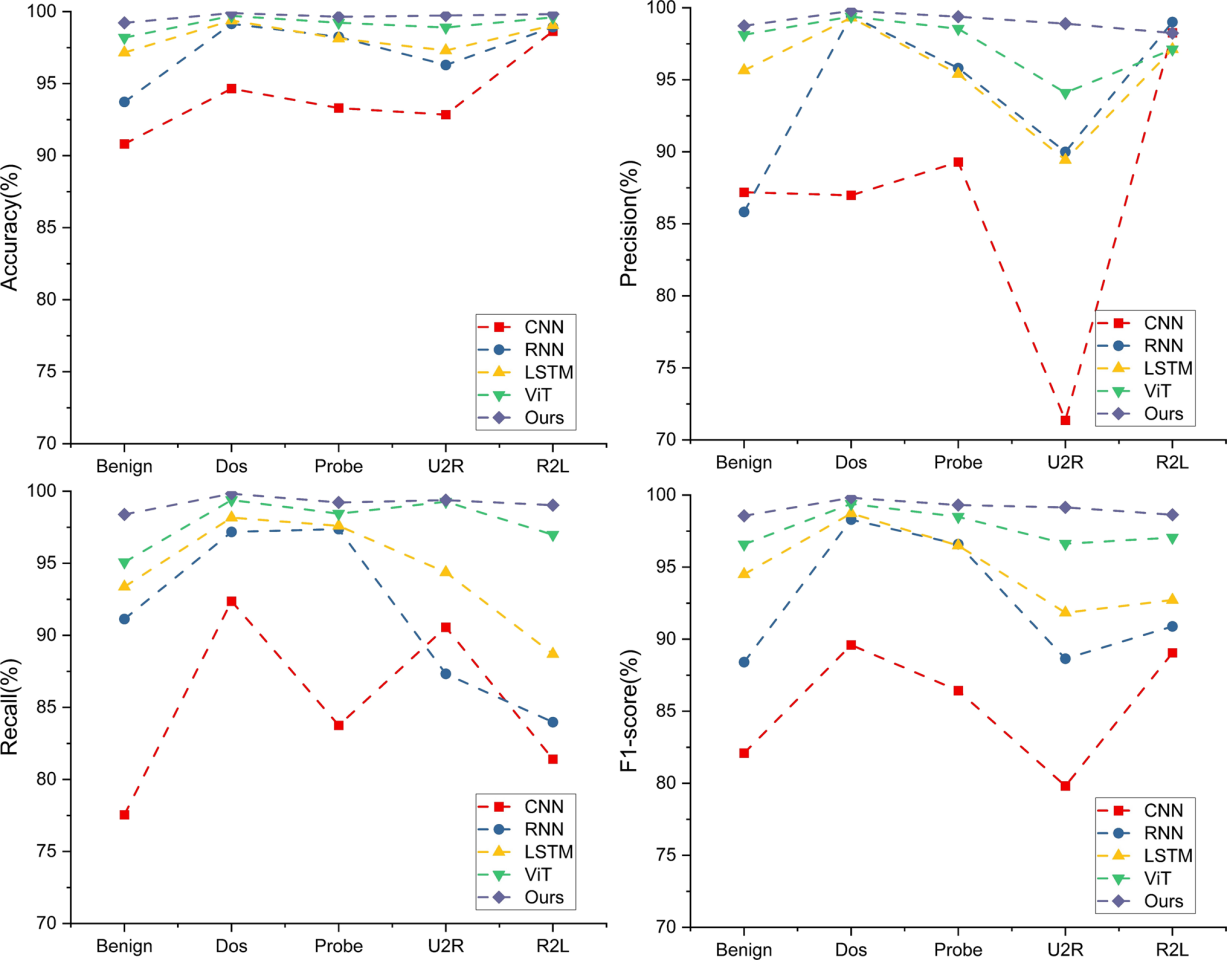
Comparison of different metrics specific to the categories of each model on NSL-KDD.

Additionally, Fig. 7A reports the comparison between IDS-MTran and some machine learning methods. It can be seen that the proposed method is second only to the J48 decision tree method used from Meena et al. ^52^, and far exceeds other machine learning methods. Furthermore, Fig. 8 reports a comparison of the metrics when specific to the attack category. Our method outperforms others on all metrics, with accuracy generally exceeding 99% and near-perfect performance on the Dos and U2R categories. The F1-score, as the reconciled average of precision and recall, are close to 99% for our method on the Normal and Dos categories, indicating that it has a well-balanced in correctly recognizing attacks as well as distinguishing types. This is crucial for real-world security applications where the nature of attacks can be diverse and unpredictable. The results clearly highlight the advantages of the proposed method, especially its robustness and reliability.

#### Comparative results on CIC-DDoS 2019

Among these datasets, CIC-DDoS 2019 is more specialized in detecting DDoS attacks, which includes a large volume of data with a comprehensive set of features. The competitors in this comparison include the RTIDS proposed by Wu et al. ^11^, the method proposed by Cil et al. ^53^ and the classical methods mentioned above.

We only conduct multiple-classification to explore the effects of each method as the number of normal traffic is small and the categories are sufficiently diverse. As shown in Fig. 7(B), compared with classical machine learning methods, the proposed method exhibit superior results, which suggests that as the complexity of such dataset increases, those conventional models may not able to find the deep non-linear relations behind. Table 3 reports the overall detection results and the proposed method still outperforms others with a considerable gap. The recall and F1-score of IDS-MTran also achieves 99.42% and 99.61%, respectively, indicating that our method is not only able to accurately identify the attacks, but also effectively cover various attack types. Additionally, Table 4 reports the quantitative results of IDS-MTran specific to attack types, which further demonstrate the robustness and advancements of the proposed method to a wide range of different attack traffic and its strong pattern coverage. Compared to the SOTA RTIDS, which also utilized the Transformer, our proposed IDS-MTran performs better and more consistently. We attribute these advantages to the multi-scale feature extraction and exploitation, which further optimizes Transformer’s ability to model traffic features.

**Table 3:** Quantitative results of multiple-classification on CIC-DDoS 2019.

Method	Accuracy	Precision	Recall	F1-score
CNN	93.27	90.73	94.21	92.44
RNN	97.64	97.45	96.39	96.92
LSTM	96.98	95.77	96.21	95.99
ViT	96.71	98.44	98.32	98.38
RTIDS ^11^	98.58	98.82	98.66	98.48
Cil et al. ^53^	94.57	80.49	95.15	87.21
Ours	**99.07**	**99.81**	**99.42**	**99.61**

The values are expressed in %, and the best one is in bold.

**Table 4:** Quantitative results of our method specific to attack types.

	Accuracy		Precision		Recall		F1-score	
	RTIDS	Ours	RTIDS	Ours	RTIDS	Ours	RTIDS	Ours
Benign	**99.47**	99.05	98.79	**98.86**	**99.74**	99.40	**99.60**	99.13
DNS	97.36	**98.43**	97.00	**98.25**	97.04	**97.98**	97.18	**98.12**
LDAP	98.03	**99.31**	97.62	**99.27**	97.32	**98.50**	97.82	**98.88**
MSSQL	96.69	**99.47**	90.23	**98.90**	93.42	**97.02**	93.35	**97.95**
NetBIOS	94.03	**98.18**	**99.60**	97.64	96.79	**97.35**	96.73	**97.49**
NTP	**99.65**	98.38	**99.51**	97.65	**99.58**	99.35	**99.58**	98.49
SNMP	98.05	**98.74**	93.82	**99.06**	95.92	**97.90**	95.89	**98.47**
SSDP	91.41	**99.51**	92.00	**98.79**	85.11	**98.60**	90.03	**98.69**
TFTP	97.71	**98.04**	**99.65**	97.65	97.51	**98.39**	98.67	**98.02**
UDP	97.29	**98.55**	75.71	**97.98**	86.05	**98.04**	85.16	**98.01**
UDPLag	96.27	**98.83**	95.91	**99.45**	86.05	**99.26**	96.09	**99.35**
WebDDos	89.77	**99.42**	88.89	**98.91**	84.46	**99.39**	86.95	**99.15**

The values are expressed in %, and the best one is in bold.

#### Comparison results on UNSW-NB15

Generally, the UNSW-NB15 is considered the most challenging one in the three IDS datasets, as it includes complex, diverse, and realistic network traffic with a wide range of modern attack types, demanding more sophisticated analysis ^54^. For this data, the selected competitors include the method proposed by Hooshmand and Hosahalli ^55^, the method proposed by Potluri et al. ^56^, DRaNN proposed by Latif et al. ^57^, the DNN method proposed by Vinayakumar et al. ^58^, and the method proposed by Ashiku and Dagli ^59^. We report the overall multiple-classification results in Table 5, and the class-wise results that specific to traffic types are presented in Table 2.

**Table 5:** The overall quantitative results on UNSW-NB15 (multiple-classification task).

	Accuracy	Precision	Recall	F1-score
CNN	91.0	88.5	90.1	89.1
RNN	94.2	91.2	92.0	91.6
LSTM	95.0	94.1	92.0	93.0
ViT	95.9	97.5	96.2	96.8
DNN ^58^	65.1	59.7	65.1	58.5
Hooshmand et al. ^55^	76.3	90.4	76.1	78.2
Potluri et al. ^56^	–	–	94.9	–
DRaNN ^57^	99.5	–	99.4	–
OURS	**99.7**	**98.5**	**99.8**	**99.1**

The values are expressed in %, and the best one is in bold.

Firstly, similar to the comparison in CIC-DDoS 2019, though the results of machine learning methods, especially the SOTA J48 Decision Tree are acceptable, they are not as competitive as they are on the simpler data like NSL-KDD. When facing such complex and variable data, those basic models may not sufficiently model the relations. Next, as reported in Table 5, the proposed IDS-MTran performs on par with the current SOTA methods in multiple-classification. However, our advantage lies in the more fine-grained detection accuracy, i.e., specific to the intrusion category. As reported in Table 6, our proposed method is robust to all traffic types, while the other methods, all show performance fluctuations to some extent. Specifically, the method proposed by Hooshmand and Hosahalli ^55^, achieves 99.0% accuracy on the Analysis and Normal type, but only 10.5 on the Dos type. For the method proposed by Potluri et al. ^56^, it performs quite well on the Generic and Normal type, but in the remaining categories, it is completely undetectable in six of them. In terms of accuracy only, DNN ^58^ performs well, however, in terms of Recall, it performs mediocrely and even appears undetectable in many categories. The difference in metrics implies that the method’s performance is extremely imbalanced. Here, DRaNN ^57^ is a strong competitor, however, our proposed method still wins with higher Recall and more stable performance.

**Table 6:** Class-wise quantitative results specific to traffic types on UNSW-NB15.

	Accuracy				Recall			
	Potluri et al. ^56^	Hooshmand et al. ^55^	DNN ^58^	Ours	DRaNN ^57^	Ashiku et al. ^59^	DNN ^58^	Ours
Analysis	0.0	99.0	**99.5**	98.7	98.2	89.5	0.0	**98.5**
Backdoor	0.0	12.0	95.1	**99.1**	**98.8**	91.2	34.4	98.1
Dos	0.0	10.5	**99.4**	98.4	98.8	94.6	97.7	**99.0**
Exploits	61.8	30.0	89.9	**97.2**	98.8	94.2	1.3	**99.4**
Fuzzers	6.8	69.5	**99.9**	97.4	97.1	88.6	0.0	**99.0**
Generic	97.7	69.1	78.3	**98.6**	**99.8**	95.1	57.1	99.2
Normal	99.7	99.0	78.9	**99.9**	-	97.2	92.8	**99.7**
Reconnaisance	0.0	77.2	92.7	**97.0**	99.2	95.1	1.8	**99.7**
Shell code	0.0	85.0	**99.0**	97.5	97.8	91.6	0.0	**99.0**
Worms	0.0	76.9	**98.8**	98.6	98.1	89.8	0.0	**99.4**

The values are expressed in %, and the best one is in bold.

On this more difficult dataset, the proposed method further demonstrates its power, maintaining high accuracy while having stable performance with minimal fluctuations. We attribute this result to the development of multi-scale architecture, complemented by the deep utilization of information at different scales, which, together with the self-attention mechanism, makes IDS-MTran an even better choice.

#### Comparison results on detection efficiency

In the practical application of IDS, detection efficiency is also a major consideration, as timely detection allows administrators to respond swiftly, thus avoiding greater damage. In this section, we conduct experiments to compare the detection efficiency. Specifically, we analyze the efficiency by recording the time taken by the model to predict each traffic sample. We report the inference speed (Frame Per Second, FPS) of each model on different datasets in Table 7.

**Table 7:** Inference speed (FPS) comparison results of different models.

	NSL-KDD	CIC-DDoS 2019	UNSW-NB15	Average
CNN	84.51	79.82	80.55	81.63
RNN	75.14	72.08	73.33	73.52
LSTM	70.52	61.71	60.10	64.11
ViT	49.11	45.07	44.72	46.30
ours	60.44	57.10	58.29	58.61

As reported, the proposed IDS-MTran achieves an average FPS of 58.61, i.e., it can achieve a good real-time performance of detecting about 58 traffic samples per second on the experimental equipment. Compared to the other models, CNN with the simplest structure has the best efficiency with an average FPS of 81.63, while RNN and LSTM with a recurrent structure achieve an average FPS of 73.52 and 64.11, respectively.The ViT model, which also uses Transformer, has a higher computational effort due to its stacked encoder structure, and only achieves an average FPS of 46.30.

### Ablation studies

#### Ablation of the loss function

**Fig. 9:**
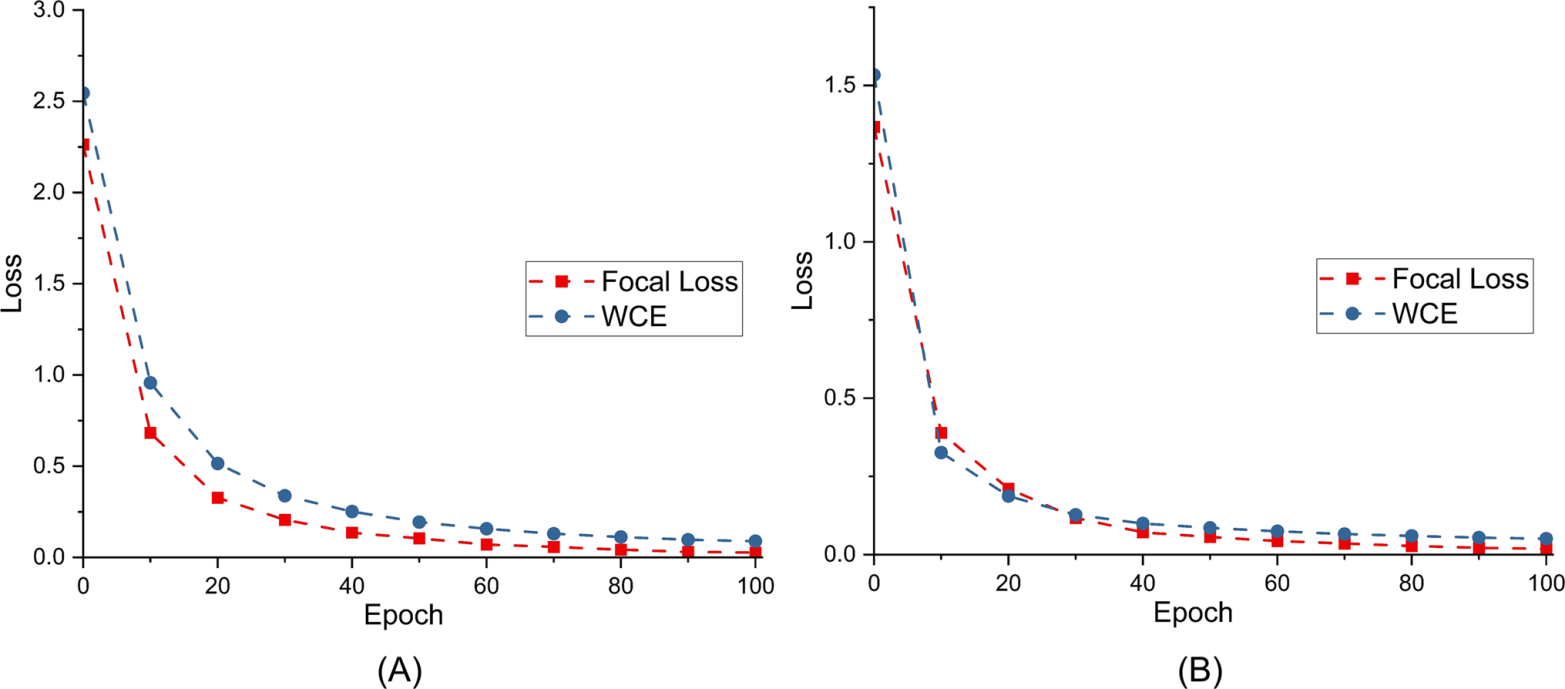
Loss changing using different functions. (**A**) Training on NSL-KDD. (**B**) Training on CIC-DDoS 2019.

To mitigate the effect of data imbalance on model training, we use Focal Loss to train IDS-MTran. This section conducts experiments to evaluate the benefits that Focal Loss brings. As shown in Fig. 9, the introduction of Focal Loss reduces the bias for both datasets, which means that it helps the model to focus on all classes without ignoring the few attacks that are difficult to classify. Meanwhile, the proposed model can converge quickly and smoothly no matter which loss function is used, indicating that it can effectively and comprehensively learn the features in training data.

#### Ablation of the multi-scale architecture

Next, we conduct ablation experiment to evaluate the effectiveness of the proposed multi-scale architecture. In this investigation, the CFE is removed, and the backbone network is connected to three linear layers to directly output the result. We separately use the three branches to perform five-classification and binary-classification task on NSL-KDD, CIC-DDoS 2019 and UNSW-NB15, respectively. Tables 8 and 9 report the results, with $P_1$, $P_2$, $P_3$ representing branches with low-, intermediate- and high-level features.

**Table 8:** Ablation results of different scales on NSL-KDD (five-classification task).

		Accuracy	Precision	Recall	F1-score
Benign	$P_1$	97.43	95.56	96.45	96.00
	$P_2$	98.02	97.33	95.24	96.27
	$P_3$	**98.81**	**98.08**	**97.55**	**97.81**
Dos	$P_1$	**99.61**	**99.08**	**99.35**	**99.21**
	$P_2$	99.01	96.92	97.88	97.40
	$P_3$	97.39	96.15	95.41	95.78
Probe	$P_1$	**99.17**	**99.39**	**97.51**	**98.44**
	$P_2$	99.00	98.09	96.27	97.17
	$P_3$	97.91	98.13	96.62	97.37
U2R	$P_1$	96.32	95.51	97.25	96.37
	$P_2$	98.81	96.49	**99.08**	**97.77**
	$P_3$	**99.41**	**97.88**	97.38	97.63
R2L	$P_1$	97.57	94.19	95.36	94.77
	$P_2$	**99.59**	**97.82**	**98.02**	**97.92**
	$P_3$	98.61	95.90	96.09	95.99

The values are expressed in %, and the best one is in bold.

**Table 9:** Ablation results of different scales on CIC-DDoS 2019 and UNSW-NB15 (binary-classification task).

	Scale	CIC-DDoS 2019				UNSW-NB15			
		Accuracy	Precision	Recall	F1-score	Accuracy	Precision	Recall	F1-score
Benign	$P_1$	98.22	97.68	98.55	98.11	97.53	97.16	98.03	97.59
	$P_2$	98.89	98.91	99.05	98.98	98.28	98.37	98.65	98.51
	$P_3$	**99.97**	**99.05**	**99.78**	**99.41**	**99.42**	**99.25**	**99.46**	**99.35**
Malicious	$P_1$	**99.86**	**99.24**	**99.51**	**99.37**	**99.18**	**99.04**	**99.21**	**99.12**
	$P_2$	99.05	98.62	98.71	98.66	98.79	98.36	98.19	98.27
	$P_3$	97.26	96.55	97.01	96.78	97.14	96.37	96.82	96.59

The values are expressed in %, and the best one is in bold.

As reported,different branches have their own focuses in capturing network traffic features. For example, the detection of normal traffic does not need to pay excessive attention to the detailed features as they usually do not have obvious abnormal patterns. Branches with higher-level features ($P_3$) can confirm the normalcy of the traffic on a macro level and determine whether the traffic is within the normal behavior, thus achieving the best performance. On the other hand, branches with lower-level features ($P_1$) are better at detecting malicious ones. For example, DoS attacks are usually launched in a short period of time through a large number of requests, Probe attacks try to obtain information about the server, and the detection requires fine-grained analysis, where $P_1$ branches perform better.

#### Qualitative analysis of the multi-scale architecture

**Fig. 10:**
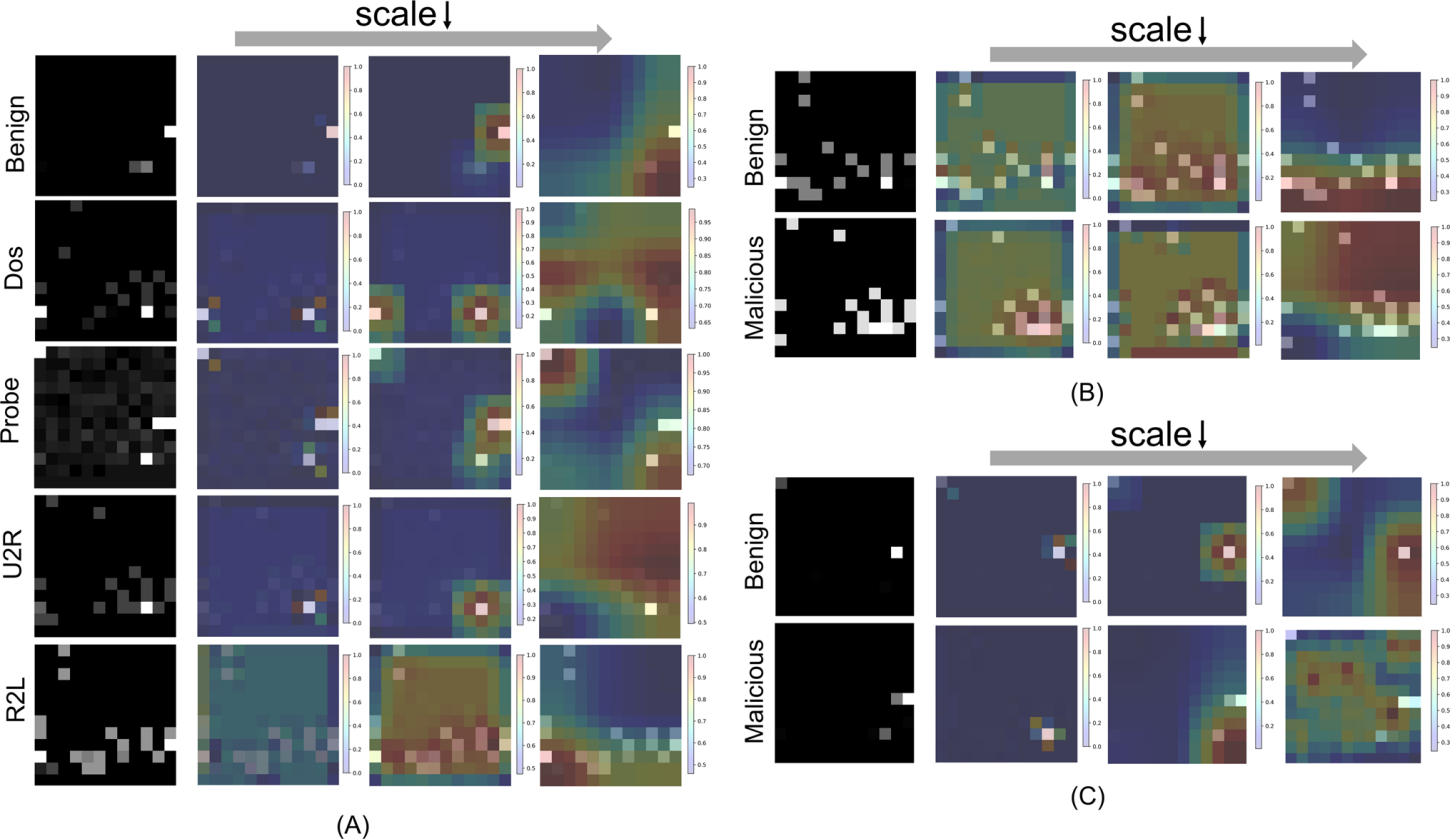
Qualitative analysis of different branches on (**A**) NSL-KDD, (**B**) CIC-DDoS 2019, and (**C**) UNSW-NB15.

To further analyze the multi-scale postulations in the proposed method, we conduct the qualitative analysis to validate. Specifically, we visualize the processing of the input at each scale on the three datasets. As shown in Fig. 10, Larger values, i.e., darker colors, indicate a higher level of attention here, which is the most helpful for classification.

As expected, the $P_1$ branch provides a fine-grained view of the traffic data with a more pronounced detail texture, focusing on localized feature variations. The small-scale patterns in this branch help to detect detailed, immediate features such as packet size variations and transmission frequency. However, there are limitations to this microscopic advantage, such as the R2L category in Fig. 10A. Its focus on features is too scattered to combine all features for a comprehensive judgment.

In contrast, the $P_3$ branch demonstrates a broader, more dispersed pattern that encompasses long-term trends and behaviors in traffic data that may deviate from the benign. More intuitively, this branch tends to have a large area of interest. It focuses on the most salient features and radiates more locations to be considered in aggregate, allowing it to perceive deviations from a global perspective and use this as a cornerstone to give macro-level results.

The intermediate $P_2$, which is larger than $P_1$ and smaller than $P_3$, integrates detailed features and general trends, blends local variations and broad patterns, and shows a comprehensive capture of attack characteristics. It provides an intermediate level of perspective that helps bridge the gap between micro-detail and macro-trends.

The combination of three scale branches then provides a robust multi-dimensional feature space. By combining micro- and macro-features, it can provide a balanced perspective, ensuring that the model can both capture the transient signals and recognize anomalous trends, providing strong support in the face of different types and complexities of attacks.

#### Ablation of the backbone

To further explore the potential factors that can enhance the performance of IDS-MTran, we ablate the Transformer-based backbone network in this section, i.e., we explore the performance in the presence of different stacking hyperparameters.

As reported in Table 10, IDS-MTran achieves the best results when the backbone stacked is 2. With the number of backbone increasing, the features become more and more abstract, and some information may be lost in the gradual compression, which can be detrimental to detecting intrusions. In contrast, when there is only one Transformer encoder, i.e., a stack of 1, the model does not perform as well, implying that the extracted features may be insufficient.

**Table 10:** Ablation results of the hyperparameters of backbone.

Stacked number	NSL-KDD		CIC-DDoS 2019		UNSW-NB15	
	Accuracy	Recall	Accuracy	Recall	Accuracy	Recall
1	98.7	98.9	98.5	97.9	98.6	98.9
2	**99.2**	**99.2**	**99.1**	**99.4**	**99.7**	**99.8**
3	98.3	98.4	98.2	98.5	99.1	98.8
4	98.1	97.9	98.3	98.2	98.7	98.6
5	97.8	97.6	97.9	97.6	98.1	97.9
6	97.1	96.8	97.3	97.1	97.4	97.2

The values are expressed in %, and the best one is in bold.

#### Ablation of the multi-scale integration

How to efficiently utilize multi-scale features is another issue. The proposed method uses CFE to process, and the results are obtained through cross-enhancement. To explore the its effect, we further conduct ablation experiments. Specifically, we set up a control group: three scales of features are directly concated and the results are obtained using three linear layers. Table 11 shows the results of the two sets of experiments. Cross-enhancement brings about 2% improvement to the Accuracy, thanks to the full utilization of different scales, it can fully explore and emphasize some easily overlooked features, thus improving the overall detection rate and making the model more robust.

**Table 11:** Ablation results of CFE.

Dataset	CFE	Accuracy	Precision	Recall	F1-score
NSL-KDD		97.92	98.82	98.19	98.50
	$\checkmark$	99.16	99.01	99.17	99.09
CIC-DDoS 2019		97.11	98.07	97.29	97.68
	$\checkmark$	99.38	99.17	98.96	99.06
UNSW-NB15		96.98	97.12	98.02	97.57
	$\checkmark$	99.74	98.49	99.78	99.13

The values are expressed in %.

## Conclusions

Aiming at the problems of under-utilization of features and poor multiple-classification accuracy in existing IDSs, this paper proposes a novel multi-scale framework IDS-MTran. It creates multi-scale branches based on the original data and leverages Transformer as the backbone to extract features. In it, the proposed PwP module effectively enhances the features and compensates the structural information, and the CFE module provides effective enhancement of feature fusion to further improve the detection accuracy. Both qualitative analysis and ablation studies prove the effectiveness of the proposed method: different scales can focus on different types of attacks, and the fused multi-scale is more robust and accurate. At the same time, sufficient comparison experiments show that IDS-MTran outperforms the existing methods in all aspects and is more suitable for real-world applications to accurately detect the attack types. The next research direction is to consider the efficient deployment of IDS-MTran to further maximize its value.

## Data Availability

The datasets analyzed in this study are available at [https://github.com/HoaNP/NSL-KDD-DataSet],[https://www.unb.ca/cic/datasets/ddos-2019.html] and [https://research.unsw.edu.au/projects/unsw-nb15-dataset].
